# Supplementary material for: Forage conservation is a neglected nitrous oxide source
Source: PNAS Nexus. 2024 Sep 24;3(9):pgae373. doi: 10.1093/pnasnexus/pgae373 (PMC11440176; doi:10.1093/pnasnexus/pgae373)
Supplement: pgae373_Supplementary_Data [file pgae373_supplementary_data.pdf]

PNAS Nexus

Supporting Information for

Forage conservation is a neglected nitrous oxide source

Seongmin Yang, Maheen Mahmooda, Rudra Baral, Hui Wu, Marc Almloff, Lauren E. Stanton, Doohong Min, Brenda K. Smiley, J. Chris Iiams, Jisang Yu, and Jeongdae Im

Jeongdae Im
E-mail: jeongdaei@ksu.edu

This PDF file includes:

Figs. S1 to S4
Tables S1 to S2
SI References

Table S1. Least square means for N_2O emissions (g-CO_2 eq. per $kg_{DM\text{-}forage}$) with standard error (SE) of the two-way analysis of variance (ANOVA) model under variance heterogeneity.[†, ‡, #]

		Treatments		
Crops	I^-	I^+	$I^+ Ch^+$	$I^+ Ac^+$
Cv_1 Hv_1	144.14 ± 10.90^a	130.33 ± 10.90^a	8.90 ± 10.90^b	114.07 ± 10.90^a
Cv_2 Hv_1	169.74 ± 10.90^a	177.72 ± 10.90^a	9.01 ± 10.90^c	93.84 ± 10.90^b
Cv_1 Hv_2	78.99 ± 7.77^a	91.82 ± 7.77^a	4.15 ± 7.77^b	71.23 ± 7.77^a
Cv_2 Hv_2	83.91 ± 7.77^a	88.79 ± 7.77^a	0.83 ± 7.77^b	78.71 ± 7.77^a
Maize	18.24 ± 1.87^a	20.57 ± 1.87^a	2.70 ± 1.87^b	16.96 ± 1.87^a
Sorghum	4.79 ± 0.22^a	2.46 ± 0.22^b	0.07 ± 0.22^c	0.52 ± 0.22^c

[a, b, c]Means in the same row with different superscript letters differ significantly ($P < 0.05$).

[†]Treatments are no inoculant (I^-), crop-specific commercial silage inoculant (I^+), inoculant and chlorate ($I^+ Ch^+$), and inoculant and acetate ($I^+ Ac^+$). The notations Cv_1 and Cv_2 indicate two alfalfa cultivars, HVX MegaTron and HybriForce 3400, respectively. The notations Hv_1 and Hv_2 indicate samples harvested at two different maturity stages, i.e., mid-bud and early-flowering, respectively.

[‡]Results are reported as lease square means ± standard error (SE) for each combination group of crop and treatment.

[#]P values of two-way analysis of variance (ANOVA) under variance heterogeneity for crop, treatment, and crop by treatment interaction are < 0.01, <0.01, and <0.01, respectively.

Table S2. Quantitative PCR primers, standards, and detection limits of each assay.

Target gene	Primer set	Primer sequences (5'-3')	Quantification range
16S rRNA [1]	8F<br>1492R	AGAGTTTGATCCTGGCTCAG<br>TACCTTGTTACGACTT	10^2-10^9
napA [2]	V67m<br>V17m	AAYATGGCVGARATGCACCC<br>GRTTRAARCCCATSGTCCA	10^2-10^9
narG [3]	narG-F<br>narG-R	TCGCCSATYCCGGCSATGTC<br>GAGTTGTACCAGTCRGCSGAYTCSG	10^2-10^9
nirK [4]	nirKC1-F<br>nirKC1-R	ATGGCGCCATCATGGTNYTNCC<br>TCGAAGGCCTCGATNARRTTRTG	10^4-10^9
nirS [5]	nirS-1F<br>nirS-6R	CCTAYTGGCCGCCRCART<br>CGTTGAACTTRCCGGT	10^3-10^9
qnorB [6]	qnorB-2F<br>qnorB-5R	GGNCAYCARGGNTAYGA<br>ACCCANAGRTGNACNACCCACCA	10^2-10^9
Clade I nosZ [7]	nosZ-1F<br>nosZ-1R	WCSYTGTTCMTCGACAGCCAG<br>ATGTCGATCARCTGVKCRTTYTC	10^2-10^9
Clade II *nosZ* [8]	nosZ 1374F<br>nosZ 1577R	CCBYTBCAYACSCARTTYG<br>TGSGASAGCTTGTTSAGSS	10^2-10^9
Bacterial - *amoA* [9]	amoA-1F<br>amoA-2R	GGGGTTTCTACTGGTGGT<br>CCCCTCKGSAAAGCCTTCTTC	10^2-10^9
Archaeal - *amoA* [10]	arch-amoAF<br>arch-amoAR	STAATGGTCTGGCTTAGACG<br>GCGGCCATCCATCTGTATGT	10^2-10^9

Abbreviations for degenerate nucleotide positions are as follows: R =A or G; K = G or T; M =A or C; S =C or G;W=A or T; Y =C or T; B =C, G, or T; D = A, G, or T; V =A, C, or G; H =A, C, or T. References for the primers and probes are included in the first column.

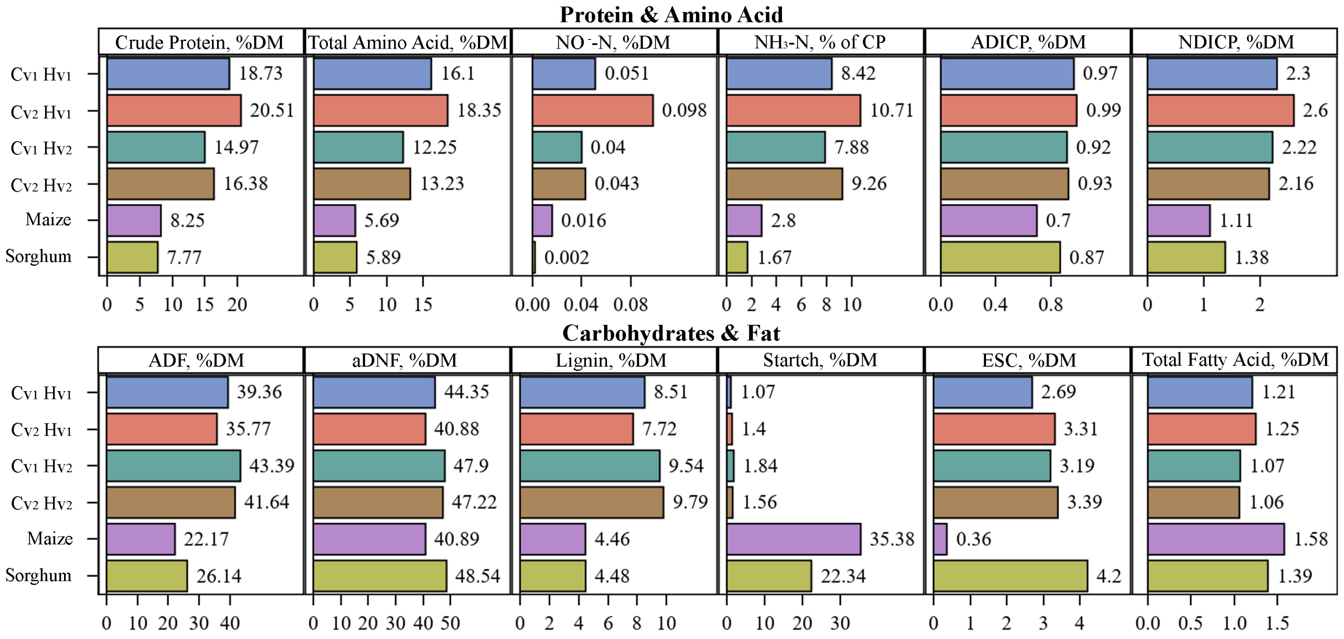

Fig. S1. Nutritional parameters of fresh maize, alfalfa (two distinct varieties harvested at two different stages of maturity), and sorghum samples. The labels Cv_1 and Cv_2 denote two alfalfa varieties, HVX MegaTron and HybriForce 3400, respectively. The labels Hv_1 and Hv_2 denote alfalfa samples harvested at the mid-bud and early flowering stages, respectively. DM: dry matter; CP: crude protein; ADICP: acid detergent-insoluble CP; NDICP: neutral detergent-insoluble CP; ADF: acid detergent-treated fiber; aNDF: amylase-treated neutral detergent fiber; ESC: ethanol-soluble carbohydrates. The numerical annotations next to the individual bars are their respective measured values.

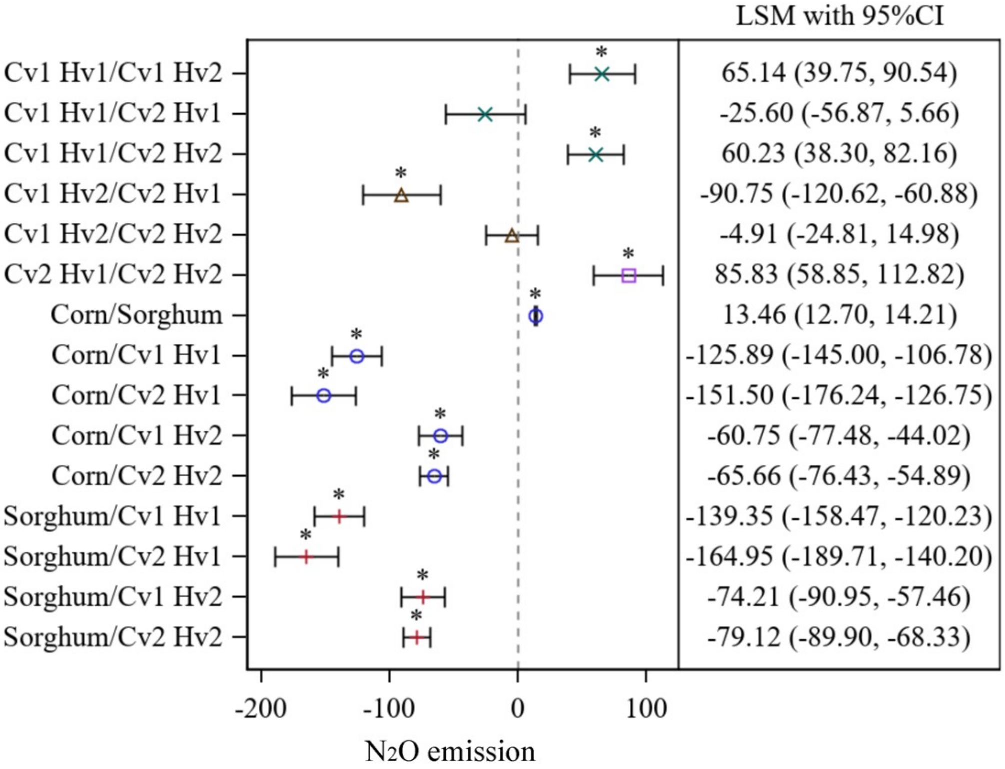

Fig. S2. Pairwise comparisons N_2O emissions for crops under the controls (I^-) group. Results are reported as lease square means for N_2O emissions with 95% confidence intervals. '*' indicates a significant difference observed between the two crop groups.

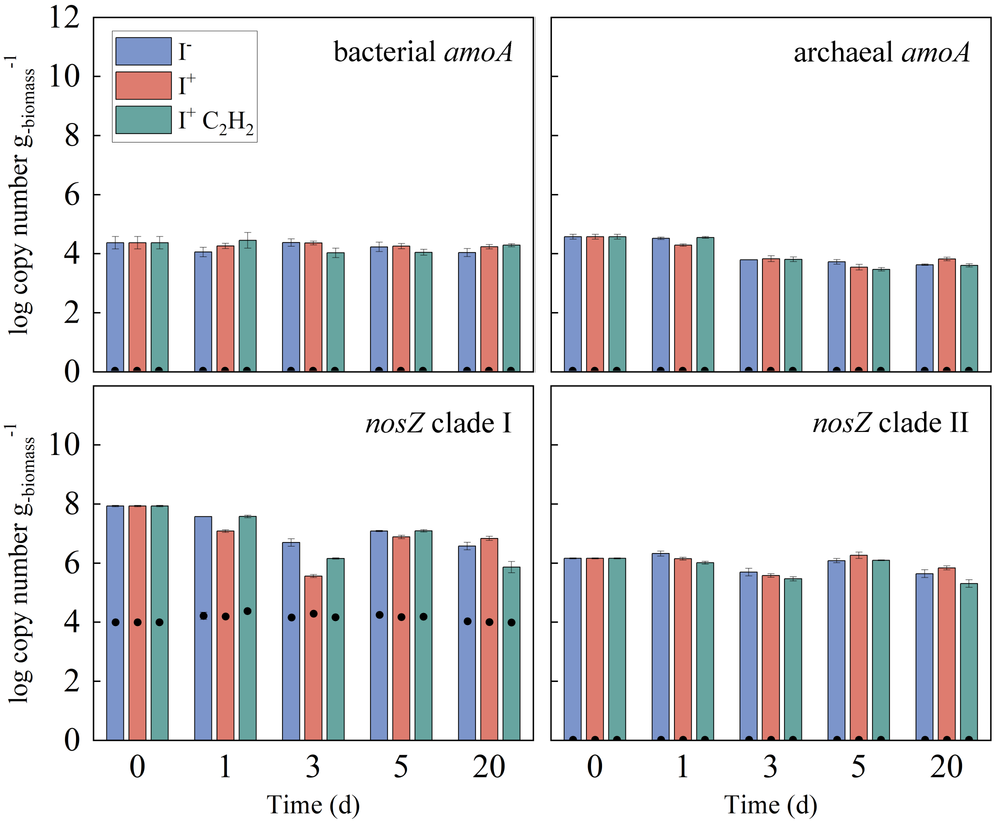

Fig. S3. Effect of acetylene (C_2H_2) on the abundance of bacterial/archaeal *amoA* genes and clade I/II *nosZ* genes (bar) and their transcripts (black circle). The error bars represent the standard deviations of the triplicate incubations. Error bars may not be visible if their magnitude is smaller than the symbols.

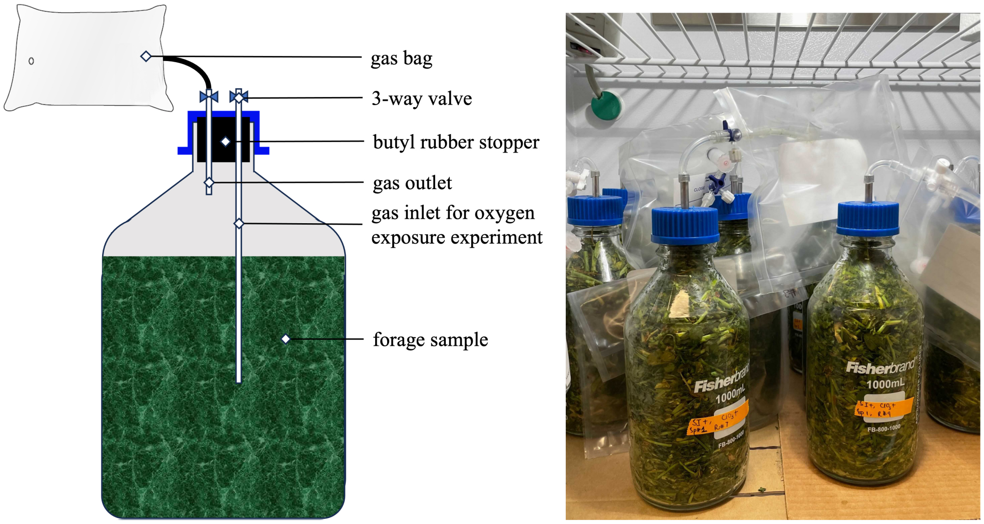

Fig. S4. A schematic (left) and photo (right) of mini-silos used in this study. The gas inlet was only used for the oxygen exposure experiment.

References

1. RI Amann, et al., Combination of 16s rrna-targeted oligonucleotide probes with flow cytometry for analyzing mixed microbial populations. *Appl. Environ. Microbiol.* **56**, 1919–1925 (1990).
2. S Henry, et al., Disentangling the rhizosphere effect on nitrate reducers and denitrifiers: insight into the role of root exudates. *Environ. Microbiol.* **10**, 3082–3092 (2008).
3. D Bru, A Sarr, L Philippot, Relative abundances of proteobacterial membrane-bound and periplasmic nitrate reductases in selected environments. *Appl. environmental microbiology* **73**, 5971–4 (2007).
4. W Wei, et al., Higher diversity and abundance of denitrifying microorganisms in environments than considered previously. *The ISME J.* **9**, 1954–1965 (2015).
5. G Braker, A Fesefeldt, KP Witzel, Development of pcr primer systems for amplification of nitrite reductase genes (nirk and nirs) to detect denitrifying bacteria in environmental samples. *Appl. Environ. Microbiol.* **64**, 3769–3775 (1998).
6. G Braker, JM Tiedje, Nitric oxide reductase (norb) genes from pure cultures and environmental samples. *Appl. environmental microbiology* **69**, 3476–83 (2003).
7. S Henry, D Bru, B Stres, S Hallet, L Philippot, Quantitative detection of the nosz gene, encoding nitrous oxide reductase, and comparison of the abundances of 16s rrna, narg, nirk, and nosz genes in soils. *Appl. environmental microbiology* **72**, 5181–9 (2006).
8. JC Chee-Sanford, L Connor, A Krichels, WH Yang, RA Sanford, Hierarchical detection of diverse clade ii (atypical) nosz genes using new primer sets for classical- and multiplex pcr array applications. *J. Microbiol. Methods* **172**, 105908 (2020).
9. JH Rotthauwe, KP Witzel, W Liesack, The ammonia monooxygenase structural gene amoa as a functional marker: molecular fine-scale analysis of natural ammonia-oxidizing populations. *Appl. Environ. Microbiol.* **63**, 4704–4712 (1997).
10. CA Francis, KJ Roberts, JM Beman, AE Santoro, BB Oakley, Ubiquity and diversity of ammonia-oxidizing archaea in water columns and sediments of the ocean. *Proc. Natl. Acad. Sci.* **102**, 14683–14688 (2005).
